# Dielectric Properties of Phosphatidylcholine Membranes and the Effect of Sugars

**DOI:** 10.3390/membranes11110847

**Published:** 2021-10-30

**Authors:** Victoria Vitkova, Vesela Yordanova, Galya Staneva, Ognyan Petkov, Angelina Stoyanova-Ivanova, Krassimira Antonova, Georgi Popkirov

**Affiliations:** 1 Georgi Nadjakov Institute of Solid State Physics, Bulgarian Academy of Sciences, 72 Tsarigradsko Chaussee, Blvd., 1784 Sofia, Bulgaria; ogikrpetkov@gmail.com (O.P.); angelina@issp.bas.bg (A.S.-I.); krasa@issp.bas.bg (K.A.); 2 Institute of Biophysics and Biomedical Engineering, Bulgarian Academy of Sciences, Acad. G. Bonchev Str., Bl. 21, 1113 Sofia, Bulgaria; v.v.yordanova.bul@abv.bg (V.Y.); gstaneva@obzor.bio21.bas.bg (G.S.); 3 Central Laboratory of Solar Energy and New Energy Sources, Bulgarian Academy of Sciences, 72 Tsarigradsko Chaussee, Blvd., 1784 Sofia, Bulgaria; popkirov@yahoo.com

**Keywords:** lipid bilayers, sucrose, capacitance, relative permittivity, dipole potential, membrane structure

## Abstract

Simple carbohydrates are associated with the enhanced risk of cardiovascular disease and adverse changes in lipoproteins in the organism. Conversely, sugars are known to exert a stabilizing effect on biological membranes, and this effect is widely exploited in medicine and industry for cryopreservation of tissues and materials. In view of elucidating molecular mechanisms involved in the interaction of mono- and disaccharides with biomimetic lipid systems, we study the alteration of dielectric properties, the degree of hydration, and the rotational order parameter and dipole potential of lipid bilayers in the presence of sugars. Frequency-dependent deformation of cell-size unilamellar lipid vesicles in alternating electric fields and fast Fourier transform electrochemical impedance spectroscopy are applied to measure the specific capacitance of phosphatidylcholine lipid bilayers in sucrose, glucose and fructose aqueous solutions. Alteration of membrane specific capacitance is reported in sucrose solutions, while preservation of membrane dielectric properties is established in the presence of glucose and fructose. We address the effect of sugars on the hydration and the rotational order parameter for 1-palmitoyl-2-oleoyl-*sn*-glycero-3- phosphocholine (POPC) and 1-stearoyl-2-oleoyl-*sn*-glycero-3- phosphocholine (SOPC). An increased degree of lipid packing is reported in sucrose solutions. The obtained results provide evidence that some small carbohydrates are able to change membrane dielectric properties, structure, and order related to membrane homeostasis. The reported data are also relevant to future developments based on the response of lipid bilayers to external physical stimuli such as electric fields and temperature changes.

## 1. Introduction

The biological significance of carbohydrate molecules and developments of their biomedical and industrial applications have stimulated intensive research toward gaining knowledge of the alteration of membrane physicochemical properties by the presence of sugars. The protection of higher plant cells from the consequences of prolonged dehydration with the help of oligo- and polysaccharides represents a prominent example of the stabilizing effect exerted by sugars on biological membranes [1,2,3]. Simple carbohydrates represent one of the major protecting excipients in preservation of cells during freeze-drying [4]. The cryoprotective efficiency of sugars is related to the molecular mechanisms of their interaction with lipid membranes. It has been established that even under conditions of tight lipid packing, polysaccharides are able to sustain the liquid-crystalline lamellar phase of phospholipids by penetrating between the lipid headgroups [5], which is coherent with their strong drought-protective role in cellular membranes [1]. Simple carbohydrates (mono- and disaccharides) are also found to exert a protective role against abnormal temperatures and dehydration in biomembranes and synthetic lipid bilayers [6]. Some disaccharides such as trehalose and sucrose are identified as efficient cryoprotectants counteracting the water deficiency via replacement of water molecules at the membrane surface [1,7,8]. Large decrease of the lateral phospholipid mobility by sucrose has been evidenced by fluorescence correlation spectroscopy measurements and molecular dynamic simulations [7]. The strong influence of simple carbohydrates and oligo- and polysaccharides on the tension of lipid monolayers has also been reported [8]. Data acquired for mechanical properties of bilayer lipid stacks [9,10] and free-standing lipid membranes [11,12,13] in the presence of mono- and disaccharides outline a more complex picture on sugar–membrane interactions.

The detailed characterization of the electrical properties of biological membranes contributes to the understanding of the governing mechanisms involved in cellular processes such as transmission of electrical impulses or synaptic activity. Research in this direction is relevant to numerous electroporation-based applications in drug delivery, electrochemotherapy, cell–cell hybridization, food processing, etc. [14]. The lipid bimolecular matrix represents the main structural entity of cell membranes. Since it is impermeable to ions, the lipid bilayer behaves as a capacitor, strongly influencing the electric field distribution in the cell. The quantification of the lipid bilayer capacitance and the estimation of its dependence on external factors allows for evaluating the charging time of membranes and membrane–field interactions, including transmembrane potential difference [15]. The alteration of the bilayer electrical capacitance in sucrose-containing solutions reported recently [16] emerged various questions regarding the effect on membrane electrical properties of dissolving simple carbohydrates in the aqueous surroundings.

Considering the close relation of the specific capacitance to membrane composition, its structure and physical state [17], we probe here the susceptibility of this important electrical parameter of lipid bilayers to the presence of sugar molecules in the aqueous surroundings. The effect of glucose, fructose and sucrose on the degree of hydration and rotational order parameter of lipid molecules is measured with regard to expected alterations of membrane dielectric permittivity [16,18,19,20]. The specific capacitance of free-standing membranes is measured by frequency-dependent electrodeformation of giant unilamellar lipid vesicles (GUVs) and Fast-Fourier transform electrochemical impedance spectrometry (FFT-EIS) of planar bilayer lipid membranes (BLMs) for acquisition of the impedance spectrum (1 Hz–50 kHz) in a couple of seconds. The simplest physical model of biological membranes is the lipid bilayer [21]. Characterized by diameters in the same range as the typical cell sizes (5–100 µm), GUVs are considered as a basic physical model of biomembranes allowing for investigation of membrane-related phenomena at the scale of single vesicles [22,23]. The elaborated experimental protocols for preparation of GUVs offer a good control of membrane composition and the physicochemical parameters of the aqueous environment. Membrane dipole potential, the degree of hydration and rotational order parameter of lipid molecules are studied in sugar-containing aqueous solutions by fluorescence spectroscopy of large unilamellar vesicles (LUVs), which are characterized by orders of magnitude smaller diameters (~100 nm) compared to GUVs. In addition to the application for modeling biomembranes, lipid vesicles are also recognized for their usage as drug and gene carriers [24], which foregrounds the investigation of their membrane physical properties and stability.

Here, we study the alteration of dielectric properties, the degree of hydration, the rotational order parameter and dipole potential of lipid bilayers in the presence of simple carbohydrates. For the first time, the effect of glucose and fructose is probed on the impedance characteristics of biomimetic lipid membranes. The reported results provide knowledge of sugar–membrane interactions in relation to the bilayer molecular organization, electrical capacitance, resistance and dielectric permittivity. The acquired new data about the alteration of the dielectric properties and molecular organization of lipid bilayers in sugar solutions are expected to facilitate the selection of the best carbohydrate to meet requirements of applications related to the response of lipid bilayers to external electric fields.

## 2. Materials and Methods

### 2.1. Materials

Synthetic monounsaturated phospholipids 1-palmitoyl-2-oleoyl-*sn*-glycero- 3-phosphocholine (16:0-18:1 PC, POPC) and 1-stearoyl-2-oleoyl-*sn*-glycero- 3-phosphocholine (18:0-18:1 PC, SOPC) purchased from Avanti Polar Lipids Inc. (Alabaster, AL, USA) in powder, are used to produce model bilayer systems as described further. Molecular probes for fluorescence spectroscopy measurements, namely 6-dodecanoyl-N, N-dimethyl-2-naphthylamine (Laurdan), 1,6-diphenyl-1,3,5-hexatriene (DPH) and 4-(2-[6-(Dioctylamino)-2-naphthalenyl] ethenyl)-1-(3-sulfopropyl)pyridinium inner salt (di-8-ANEPPS) are purchased from Sigma-Aldrich (Darmstadt, Germany). The same provider supplied chloroform and methanol for lipid solutions, sodium chloride for aqueous solutions as well as D-(+)-Glucose (C_6_H_12_O_6_, BioXtra, ≥99.5%, GC), D-(−)-Fructose (C_6_H_12_O_6_, BioUltra, ≥99.0%, HPLC) and sucrose (C_12_H_22_O_11_, BioXtra, ≥99.5%, GC). Pentane CH_3_(CH_2_)_3_CH_3_ and hexane CH_3_(CH_2_)_4_CH_3_ of HPLC grade are purchased from Honeywell, Riedel-de Haën (Seelze, Germany). Polydimethylsiloxane (PDMS) is provided by Dow Corning (Midland, MI, USA). All substances used for the preparation of bilayer membranes and vesicle suspensions are utilized as purchased. Aqueous solutions are prepared with bidistilled water from a quartz distiller.

### 2.2. Methods

#### 2.2.1. Preparation of Giant Unilamellar Vesicles

Electroformation method is applied to produce giant unilamellar vesicles (GUVs) for measurements of membrane electrical capacitance. The electroformation cell consists of two indium tin oxide (ITO)-coated glass plates, serving as electrodes, which are separated by a PDMS spacer [25]. A small amount (~50 μg) of POPC or SOPC with lipid concentration of 1 g/L in chloroform-methanol solvent (9:1 volume parts) is uniformly spread on the ITO-coated side of each glass plate. After the complete evaporation of the organic solvents achieved under vacuum, the electroformation chamber is filled with 1 mmol/L NaCl aqueous solution, which is sugar-free (control) or contains up to 300 mmol/L of glucose, fructose or sucrose. AC electric field with 10 Hz frequency and peak-to-peak voltage amplitude successively increased to 4 V is applied to the chamber. A high yield of quasispherical unilamellar vesicles with diameters of dozens of micrometers is obtained in several (~3) hours. The conductivities of the aqueous solutions are measured with Cyberscan PC510 (Eutech Instruments, Singapore). Prior to electrodeformation measurements of GUVs, we add 0.1 mmol/L of sodium chloride to the suspension in order to increase the conductivity of the external solution in accordance with the requirements of the GUV electrodeformation method (see Section 2.2.5).

#### 2.2.2. Preparation of Large Unilamellar Vesicles

Fluorescence spectroscopy of 6-Dodecanoyl-N,N-dimethyl-2-naphthylamine (Laurdan), 1,6-diphenyl-1,3,5-hexatriene (DPH) and Di-8-ANEPPS is performed on large unilamellar vesicles (LUVs). LUVs are formed by means of the extrusion method as described in [26]. The lipid and the indicated fluorescent probe are dissolved and mixed in chloroform/methanol (1:1 v/v). Laurdan or DPH are mixed with the lipids in the initial organic solution at 1:200 probe:lipid molar ratio. The fluorescent probe di-8-ANEPPS in ethanol at 1 mg/mL stock solution concentration, is mixed with the lipids in the initial organic solution at 1:250 probe-to-lipid molar ratio. Afterward, the solvent is removed under a stream of oxygen-free dry nitrogen. The residues are subsequently maintained under vacuum overnight, and then filtered (0.2 µm) sugar solution is added at room temperature (22 °C) to yield a lipid concentration of 1 mM. The samples are heated at 60°C for 5 min, vortexed for 1 min, then left in a sonication bath for 1 min, and finally cooled in ice for 5 min. This heating/cooling procedure is repeated three times to ensure the sample homogenization. The multilamellar vesicles obtained at this stage are then extruded with a LiposoFast small-volume extruder equipped with polycarbonate filters (Avestin, Ottawa, Canada) as follows: 11 extrusions through 800 nm, followed by 21 extrusions through 100 nm filters. The final lipid concentration in cuvette is 200 µmol/L. LUV samples are studied the same day after equilibration for 20 min at room temperature (22 °C). Each sample is measured 10 times after gentle pipetting and averaged by three different LUV preparations.

#### 2.2.3. Preparation of Bilayer Lipid Membranes

Bilayer lipid membranes (BLMs) are prepared after Montal and Mueller [27,28] in BC-20A chamber (Eastern Scientific LLC, Rockville, MD, USA), consisting of two similar Teflon blocks, each one with volume 2 mL, designed to hold 0.025 mm thin Teflon film in between. Solvent-free membrane patch is generated across a hole in the Teflon film with diameter 100 µm following the procedure described in [27]. The first step consists in pre-painting each side of the hole with ~1 μL of 1 wt% hexadecane in pentane and allowing the solvent to evaporate for ~5 min. The next step consists in pipetting in each compartment 0.5 mL of the aqueous solution containing 1 mM NaCl for the control sample or 1 mM of NaCl and 200 mM of glucose, fructose or sucrose. Thereafter, 15 μL of freshly prepared membrane-forming lipid solution is spread on top of the buffer in both compartments in order to ensure the presence of several lipid layers on the air-water interphase. The gentle addition of 1.5 mL of the corresponding aqueous solution consequently in each part of the cuvette leads to the generation of POPC bilayer on the aperture of the Teflon partition between the two compartments [28]. The final control of membrane formation and quality is performed electrically [27]. EIS-FTT measurement is effectuated immediately afterward.

#### 2.2.4. Fluorescence Spectroscopy of Laurdan-, DPH- and Di-8-ANEPPS-Labeled LUVs

The lipid structural order parameter of the bilayer in the fatty acid core and at the glycerol level are probed by fluorescence spectroscopy of DPH [29] and Laurdan [30], respectively. The excitation wavelength for Laurdan is 355 nm. In disordered lipid surroundings, its emission maximum with intensity $I_{490}$ is centered at 490 nm. In more ordered lipid environment, the maximum of Laurdan emission is shifted at 440 nm with intensity $I_{440}$. The lipid packing is quantified by the parameter GP representing Laurdan generalized polarization accordingly [30]:

(1) $$GP=\frac{I_{440}-I_{490}}{I_{440}+I_{490}}$$

It theoretically assumes values from −1, corresponding to disordered membrane up to 1 for most ordered molecules. We record three times all emission spectra from 390 to 600 nm, then average, and subtract background. For every studied LUV suspension, the emission spectrum of Laurdan is obtained and GP values are calculated in the temperature range (20−60) ℃.

We apply 1,6-diphenyl-1,3,5-hexatriene (DPH) fluorescence spectroscopy [31] to assess membrane fluidity as a function of sugar concentration in LUV samples. DPH represents a hydrocarbonic probe, which is nearly non-fluorescent in water. Contrastingly, in lipid membranes, it exhibits strong fluorescence. In our experiment, the excitation is adjusted to 358 nm. We record the emission at 430 nm. The fluorescence polarization is measured as described in [32]. The DPH fluorescence anisotropy $r_{DPH}$, quantifying membrane fluidity is expressed by the intensities I of the polarized components of the fluorophore emission:

(2) $$r_{DPH}=\frac{I_{VV}-GI_{VH}}{I_{VV}+2GI_{VH}},$$

where V and H stand for “vertical” and “horizontal”, corresponding to the orientation of the polarization axis with respect to the light direction. The first index indicates the polarization of the excitation light and the second one denotes the polarization of the emission signal, corresponding to the orientation of the excitation and emission polarizers, respectively. The grating factor $G=I_{HV}/I_{HH}$ reflects the sensitivity of the instrument toward vertically and horizontally polarized light. Around the main phase transition of the bilayer, the fluorescence anisotropy of DPH sharply increases. It can assume values from −0.2 to 0.4 [31].

The membrane dipole potential in sugar-containing aqueous solutions is quantified by the fluorescence excitation ratio of the potential-sensitive fluorescent styryl dye di-8-ANEPPS. In the case of di-8-ANEPPS incorporated into LUVs, fluorescence is excited at 420 nm and 520 nm and detected at 670 nm. It has been shown that the fluorescence intensity ratio $R_{\mathrm{ex}}=I_{670\;\left(\mathrm{exc}.420\right)}/I_{670\;\left(\mathrm{exc}.520\right)}\;$is proportional to the dipole potential $\Psi_{d}$, independently of fluidity effects [33,34,35]:

(3) $$\Psi_{d}=\left(R_{\mathrm{ex}}+0.3\right)/0.0043$$

The dipole potential occurs transversally between the water–lipid interface and the hydrocarbon interior from the contribution of all polarized and polarizable chemical groups of lipid molecules and by the hydration shell of the bilayer [36].

FP-8300 spectrofluorimeter (Jasco) equipped with polarizers and a thermostatted (±0.1 ℃) cuvette holder with quartz cuvettes is used. Samples are equilibrated for 5 min at the desired temperature. Excitation and emission slits are adjusted to 5 nm.

#### 2.2.5. Electrodeformation of GUVs

The specific electrical capacitance, $C_{m}$, of POPC and SOPC membranes in the presence of small carbohydrates (sucrose, glucose and fructose) is measured from the frequency-dependent deformation of GUVs in alternating electric field [37]. The vesicle shape transformation has been established to depend on the ratio Λ between the conductivity $\lambda_{in}$ of the aqueous solution, enclosed by the vesicle membrane, and the conductivity $\lambda_{out}$ of the external (suspending) medium [38,39]. In the case of more conductive external medium, upon increasing the AC field frequency, a vesicle with radius r placed in a more conductive aqueous solution changes its shape from prolate to oblate with respect to the field direction. During this morphological transition, the intermediate frequency, $f_{cr}$, at which the quasispherical shape is assumed, is given by the expression [38]:

(4) $$f_{cr}=\frac{\lambda_{in}}{2\pi r{\bar{C}}_{m}}{\left[\left(1-\Lambda \right)\left(\Lambda +3\right)\right]}^{-1/2}$$

${\bar{C}}_{m}$ denotes the resultant capacitance of a series of three capacitors. These are the bare lipid bilayer, $C_{m}$, and the capacitances of the diffuse charge regions resembling electric double layers in the aqueous solution at the two sides of the bilayer, denoted by $C_{D,in}$ and $C_{D,ex}$, respectively:

(5) $${\bar{C}}_{m}={\left(1/C_{m}+1/C_{D,in}+1/C_{D,ex}\right)}^{-1}$$

On the length scale of GUVs with radii ~10 µm and bilayer thickness *d* ~ 5 nm (*d* ≪ *r*) [37] the membrane is described as a two-dimensional surface with dielectric permittivity $\varepsilon_{m}=\varepsilon_{rm}\varepsilon_{0}$, where $\varepsilon_{rm}$ stands for the relative dielectric constant of the bilayer and $\varepsilon_{0}$ ≈ 8.85 × 10^−12^ F/m is the vacuum permittivity. Therefore, the specific capacitance $C_{m}$ of the bilayer is given by:

(6) $$C_{m}=\varepsilon_{m}/d$$

The capacitance of the electric double layers is represented by the capacitance of a planar capacitor with thickness equal to the Debye length, $\lambda_{D}$, and dielectric constant equal to the dielectric constant of the aqueous solution $\varepsilon_{r}\approx$ 80 [40]. The Debye length is related to the molar concentration c of a 1:1 electrolyte by the expression $\lambda_{D}=0.303/\sqrt{c}$ nm [41]. Considering the concentrations of NaCl applied here, we estimate the capacitance for the double layers of free charges in the aqueous solution on both sides of the bilayer $C_{D,in}$ and $C_{D,ex}$. As discussed in [38], their contribution increases at lower salt concentrations as well as for high enough values of the bilayer capacitance.

It has been established that the dielectric properties of the ionic double layers near the membrane are related to the orientational ordering of water dipoles in the aqueous surroundings as well as in the headgroup region of lipid bilayers. We analyze the former in the light of the theoretical evaluations published so far [40,42,43], while the latter corroborates the necessity to investigate the effect of sugar molecules on the bilayer dielectric properties. In the case of lipid membranes composed of zwitterionic phosphatidylcholines, studied here, it has been shown that at much higher monovalent salt concentrations the relative permittivity in the dipolar headgroup region is decreased as a result from the saturation effect in orientational ordering of water dipoles [44]. In the present study, we consider PC membranes in sugar-containing electrolyte solutions with ionic strength, which is two orders of magnitude lower than in [44]. At zero surface charge density corresponding to the model lipid system studied here, the value taken for $\varepsilon_{r}\approx$ 80 represents a good evaluation for the relative permittivity of the aqueous solution surrounding the bilayer as shown in [43]. The electric field strengths applied are far below the electroporation threshold [37,45].

The electrodeformation measurements are conducted in a chamber consisting of two parallel glass slides, which are separated by a 0.5 mm-thick inert spacer (Sigma-Aldrich Inc., St Louis, MO, USA). AC electric field from an arbitrary waveform generator (33120A, HP/Agilent, Santa Clara, CA, USA) is applied to a pair of two rectangular parallel ITO-electrodes deposited on the lower inner surface of the chamber at 1 mm apart. The measurement is performed by varying the frequency of the imposed uniform field in the range of 10–200 kHz. The field strengths ≤ 7 kV/m, applied here, are two orders of magnitude lower than the electroporation threshold [37,45]. The vesicle electrodeformation is observed and recorded using a phase-contrast microscope (B-510PH, Optika, Ponteranica, BG, Italy) equipped with a dry objective (×40, 0.65 numerical aperture) and Axiocam ERc 5s camera 5 MP (Zeiss, Jena, Germany) connected to a computer for image recording and processing with resolution of 0.1 μm/pixel. The ratios 0.87 ≤ Λ ≤ 0.95 correspond to a more conductive suspending solution. We perform data analysis following the original approach of Salipante et al. [16,38].

#### 2.2.6. Fast Fourier Transform Impedance Spectroscopy of BLMs

Fast Fourier transform electrochemical impedance spectroscopy (FFT-EIS) is based on measurements in the time domain, applying a multisine perturbation signal of a small-amplitude ~10 mV peak-to-peak covering the desired frequency range of 1.5 Hz–50 kHz. The perturbation and the respective response signals are simultaneously measured and subsequently transferred to the frequency domain using the fast Fourier transformation (FFT). The major advantages of the approach chosen here are the fast acquisition of the impedance spectrum obtained in a couple of seconds and the possibility to easily recognize violation of stationarity [46,47]. Thus, the FFT-EIS method appears to be suitable for studying the impedance properties of dynamic and unstable samples such as lipid bilayers. The perturbation voltage is applied potentiostatically using a two-electrode scheme with two platinum electrodes to supply the current and to measure the voltage across membrane. The contact area of Pt electrodes with the bulk phase in both compartments of Montal–Mueller cell is large enough to guarantee that the contribution of the electrode double electric layer is negligible in the frequency range covered.

**Figure 1:**
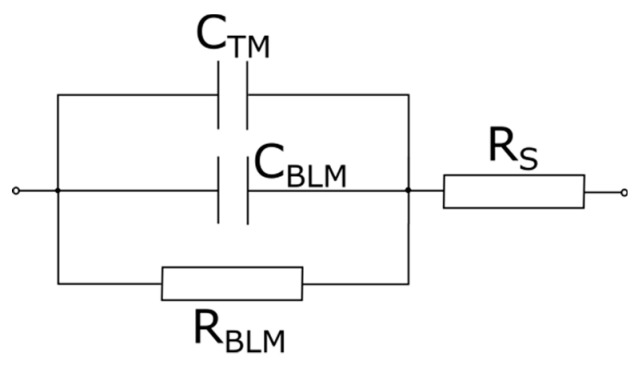
Equivalent circuit model of Montal–Mueller cell for formation of bilayer lipid membranes (BLMs): $C_{\mathrm{BLM}}$—BLM capacitance; $R_{\mathrm{BLM}}$ —BLM resistance; $C_{\mathrm{TM}}$ —capacitance of the Teflon membrane; $R_{s}$ —resistance of the aqueous solution.

The analysis of FFT-EIS data is performed by equivalent circuit modeling. From electrical point of view Montal–Mueller BLM set-up is represented by the capacitance $C_{\mathrm{BLM}}$ and resistance $R_{\mathrm{BLM}}$ of the planar lipid bilayer, connected in parallel together with the capacitance of the Teflon membrane $C_{\mathrm{TM}}$. The resistance $R_{s}$ of the surrounding solution is connected in series as depicted in Figure 1. Considering the 100 µm aperture surface area $S_{m}=$ 8.10^−5^ cm^2^ we deduce membrane-specific capacitance $C_{m}=C_{\mathrm{BLM}}/S_{m}$ and resistance $R_{m}=R_{\mathrm{BLM}}S_{m}$. The reported values are calculated as the weighted average of 4–7 independent measurements averaged over 10 repetitions each. The capacitance $C_{\mathrm{TM}}$ is measured independently as discussed below.

## 3. Results

### 3.1. Specific Capacitance of Lipid Bilayers in the Presence of Simple Carbohydrates

#### 3.1.1. Free-Standing Lipid Bilayers—Electrodeformation of GUVs

**Figure 2:**
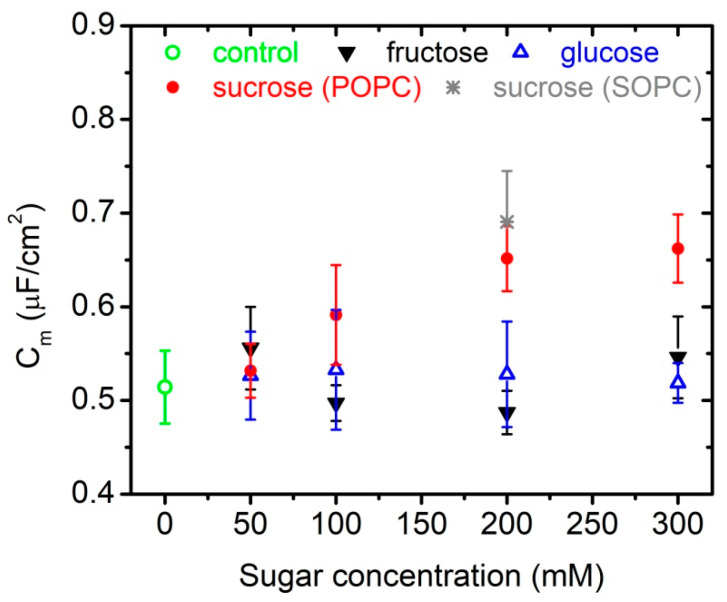
Specific capacitance of (i) POPC bilayers in the presence of 1 mmol/L NaCl and up to 300 mmol/L of glucose, fructose or sucrose; (ii) SOPC membranes in 1 mmol/L NaCl, 200 mmol/L of sucrose obtained from electrodeformation of GUVs.

The specific capacitance of POPC bilayers is measured in aqueous solutions of 1 mM NaCl containing different concentrations of glucose, fructose or glucose up to 300 mmol/L. In order to probe for specific effects, we determine the same parameter also for another type of phosphatidylcholine membranes, namely SOPC, at 200 mmol/L of sucrose in the aqueous surroundings. Our choice of synthetic lipids is motivated by their controlled chemical composition combined with the property to mimic well natural lipid extracts, e.g., egg-yolk or soybean phosphatidylcholine extracts. The values obtained for membrane specific capacitance together with the error calculated from the fit of our experimental data for every sugar concentration are summarized in Table 1 and Figure 2.

For each sugar concentration, we measure the transition frequency$\;f_{cr}$ (Equation (4)) of 6 to 15 vesicles. The acquired experimental data for $f_{cr}$ as a function of the inverse radii of vesicles are fitted for fixed $\lambda_{in}$ and Λ. Latter are the same for all recorded and analyzed vesicles in one batch. Membrane capacitance serves as a single fitting parameter. Our results show no alteration of the specific capacitance of POPC bilayers in aqueous solutions of glucose and fructose at concentrations of up to 300 mmol/L and in the presence of 1 mmol/L NaCl. In glucose solutions, we obtain $C_{m}=0.52\pm 0.02\;$µF/cm^2^ with goodness of fit 0.91. The value calculated from the data acquired in fructose solutions is $C_{m}=0.51\pm 0.01\;$µF/cm^2^ with goodness of fit 0.55. In sucrose-containing aqueous surroundings we measure 25%-higher values of membrane specific capacitance at 200 and 300 mmol/L sugar concentrations compared to the control value of the same parameter in 1 mmol/L NaCl (Figure 2). Similar increase of $C_{m}$ is reported also for SOPC bilayers at 200 mmol/L of sucrose in water.

#### 3.1.2. Suspended Planar Lipid Bilayers—FFT-EIS of BLMs

**Figure 3:**
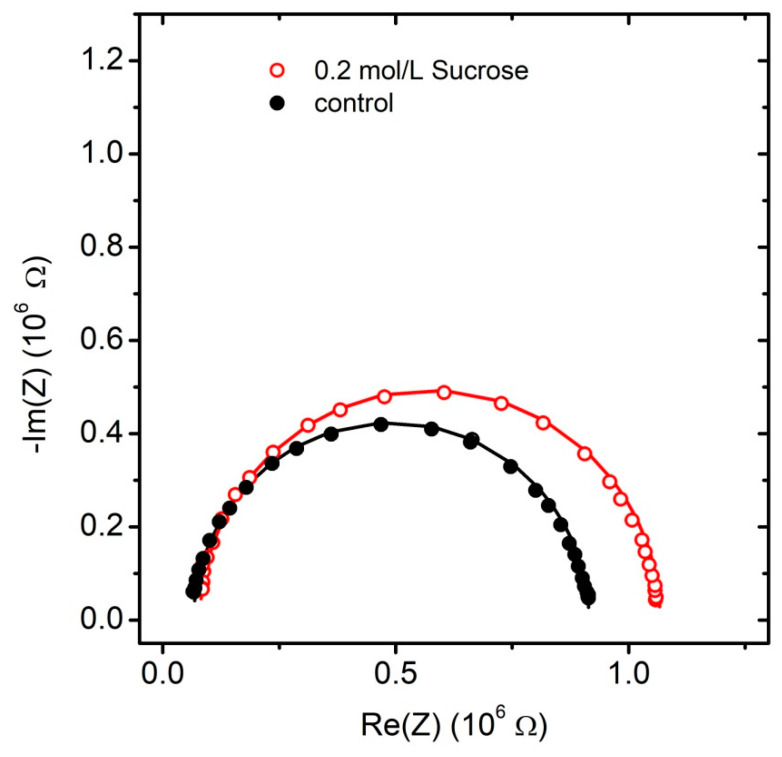
Impedance spectra plots in the complex plane (Nyquist diagrams) measured for POPC BLMs in 1 mmol/L NaCl (solid circles) and 200 mmol/L of sucrose (hollow circles). Lines represent equivalent model circuit fits.

**Figure 4:**
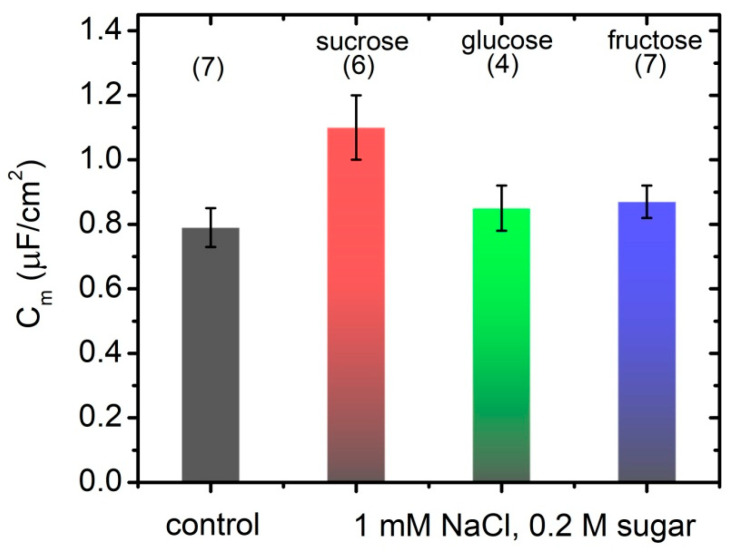
Specific capacitance of POPC BLMs in 1 mmol/L NaCl (control); 1 mmol/L NaCl and 200 mmol/L of sugar (sucrose, glucose and fructose); data acquired by FFT-EIS; $C_{m}$ values calculated as the weighted average of 4–7 independent measurements, each of them averaged over 10 repetitions.

Fast Fourier transform electrochemical impedance spectroscopy (FFT-EIS) is applied to probe the effect of simple carbohydrates on the impedance characteristics of POPC bilayer lipid membranes. Montal–Mueller technique for planar lipid bilayers yields stable bilayer membranes for assessment of their impedance properties to be probed here by an FFT electrochemical impedance spectrometer. Control samples are obtained in 1 mmol/L NaCl. Impedance measurements are performed also in the presence of 1 mmol/L NaCl and 200 mmol/L glucose, fructose or sucrose. Solvent-free BLMs are produced by Montal–Mueller technique [27,28] across a 100 µm aperture in a 0.025 mm thin Teflon film. The application of Montal–Mueller cell for the measurement of BLM electrical properties requires considering the capacitance $C_{\mathrm{TM}}$ of the Teflon membrane. The value measured for $C_{\mathrm{TM}}=56.33\pm 0.23\;$pF is comparable to the expected capacitance of the bilayer lipid membrane suspended on the hole. Hence, it has been considered in the equivalent circuit modeling as shown in Figure 1. In order to exclude any preparation-related artifacts, the impedance of at least four POPC BLMs is measured for each carbohydrate tested. Impedance spectra plots in the complex plane (Nyquist diagrams) measured for POPC BLMs in 1 mmol/L NaCl (solid circles) and 200 mmol/L of sucrose (hollow circles) with equivalent model circuit fits (lines) are displayed in Figure 3. The average values of the specific capacitance and resistance of POPC BLMs with their standard deviations obtained in 1 mmol/L NaCl aqueous solution and in 200 mmol/L sugar-containing media are summarized in Table 2 and in Figure 4.

All measurements are performed in aqueous solutions containing 1 mmol/L NaCl. The equivalent model circuit fits give for the studied samples values of the aqueous surrounding series resistance $R_{s}$ (Figure 1) ranging from 36 ± 1 to 136 ± 8 kΩ. In all aqueous solutions, we measure POPC membrane resistance $R_{m}$ ≈ 10^6^ Ω cm^2^ (cf. Table 2), which is similar to the values for lipid bilayers composed of other synthetic phosphatidylcholines as reported so far [48,49,50]. Upon addition of sugars in the aqueous phase we observe a slight increase of ~10–14% in BLM resistance as shown in Table 2.

FFT-EIS measurements of POPC BLMs impedance yield higher capacitance values compared to $C_{m}$ deduced from experiments with GUVs for the same lipid bilayer and aqueous solution compositions (cf. Table 1 and Table 2). Membrane specific capacitance remains unchanged in 200 mmol/L glucose or fructose solutions, while in the presence of the same concentration of sucrose, we obtain capacitance value almost 40% higher compared to the sugar-free control sample (cf. Table 2 and Figure 4). The latter is qualitatively consistent with the respective results for free-standing POPC membranes acquired from electrodeformation of GUVs, which have shown slightly lower increase of 25% of membrane specific capacitance.

### 3.2. Lipid Packing in the Presence of Sugars

**Figure 5:**
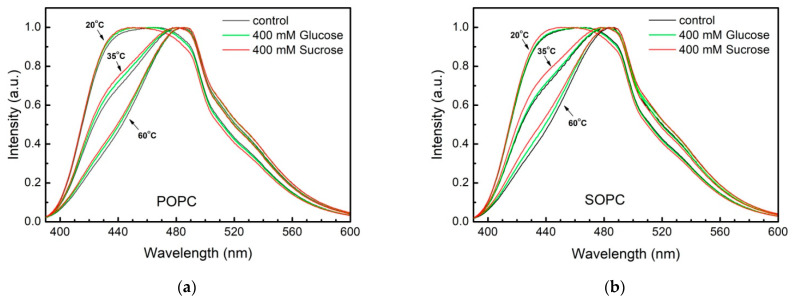
Laurdan intensity profiles from (**a**) POPC and (**b**) SOPC LUVs in water (control) and in 400 mmol/L glucose or sucrose scanned at 20, 35, and 60 °C.

We probe the effect of glucose and sucrose at concentrations in the aqueous phase up to 400 mmol/L on the lipid packing in the bilayer at various temperatures ranging from 20 to 60 °C. Normalized fluorescence emission spectra of Laurdan/SOPC and Laurdan/POPC vesicles at different temperatures in bidistilled water as well as in 400 mmol/L glucose and sucrose aqueous solutions are depicted in Figure 5. The emission spectra at 20 °C of both control and sugar-containing POPC and SOPC vesicles exhibit two peaks of nearly equal intensities centered at 430 nm (blue-shifted) and 490 nm (red-shifted), respectively. The presence of two peaks indicates that Laurdan senses two environments, one ordered and another one, more disordered, which could be associated with the presence of saturated and unsaturated fatty acids in PC molecules.

**Figure 6:**
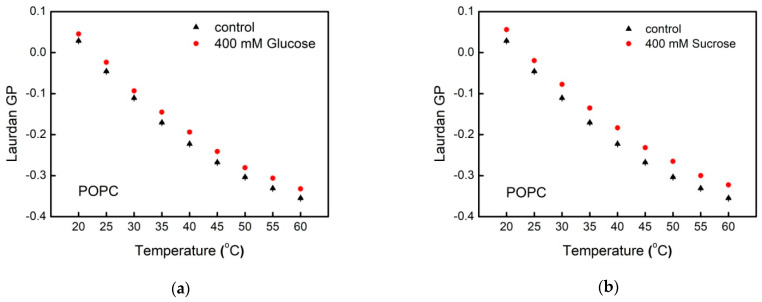

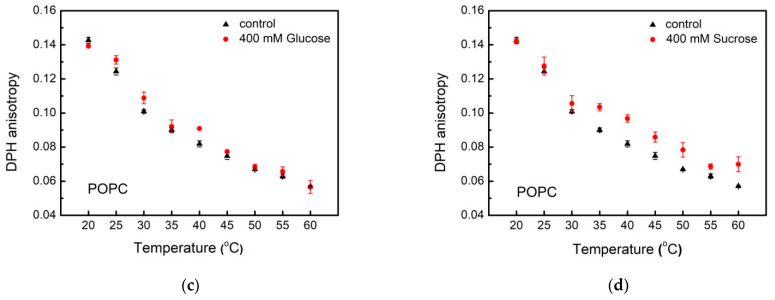
Fluorescence spectroscopy data for POPC membranes: Laurdan GP as a function of temperature in: (**a**) glucose and (**b**) sucrose solutions; DPH anisotropy vs. temperature in (**c**) glucose and (**d**) sucrose solutions.

At 20 °C the spectral profiles of POPC vesicles in sugar solutions resemble the profile of the control sample similarly characterized by two peaks centered at 430 and at 490 nm. At the same temperature, the disordered red-shifted peak is characterized with slightly lower intensity compared to the blue-shifted one in sucrose solutions both for POPC and SOPC vesicles (20 °C, red curves in Figure 6a,b). The temperature increase leads to the progressive disappearance of the more ordered blue-shifted peak reflecting that at 60 °C the mobility of the two fatty acids is undistinguishable for the fluorescent probe.

Laurdan GP values for POPC LUVs in water (control) and in sugar-containing aqueous solution as a function of the temperature are presented in Figure 6a,b. For the sake of clarity only the data for the highest sugar concentration studied are displayed in the figures. The fluorescence emission spectrum of Laurdan-labeled LUVs is measured at 20 to 60 ℃. The generalized polarization GP is calculated according to Equation (1) in the control sample as well as for the carbohydrate concentrations studied. As it is indicated above, GP scale values vary between −1 and 1. In our experiments, the control POPC LUVs exhibit GP values from 0.05 (20℃) to −0.35 (60 ℃) corresponding to intrinsically loosely packed lipids in liquid-disordered phase ($L_{d}$). A similar trend is observed for Laurdan GP values for POPC LUV suspensions in the presence of glucose and sucrose. In sugar-containing aqueous environment, we report higher GP values compared to the control samples in the whole temperature range scanned.

**Figure 7:**
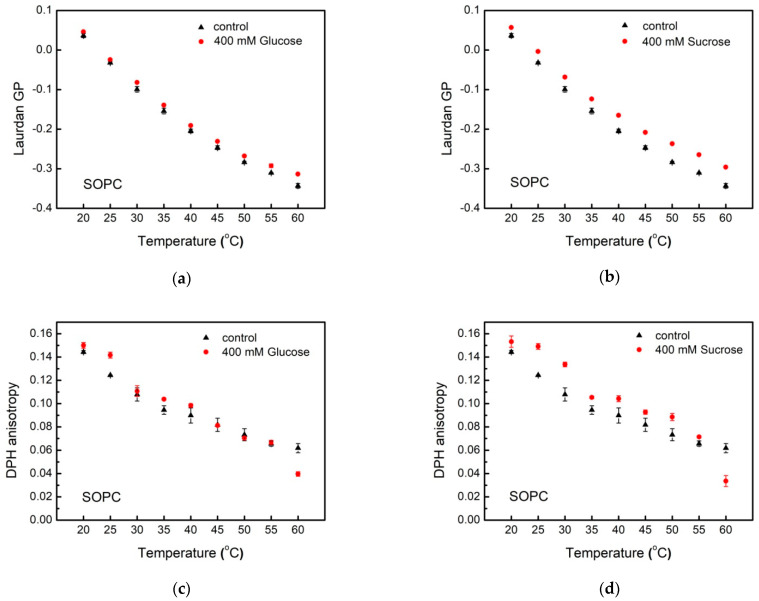
Fluorescence spectroscopy data for SOPC bilayers: Laurdan GP as a function of temperature in: (**a**) glucose and (**b**) sucrose solutions; DPH anisotropy vs. temperature in (**c**) glucose and (**d**) sucrose solutions.

We quantify the effect of glucose and sucrose on the molecular organization in membrane hydrophobic core using DPH. The fluorescence anisotropy of this molecular probe is related to the fatty acids mobility [31]. The steady-state DPH anisotropy, $r_{DPH}$, within the bilayer is determined according to Equation (2). As discussed above, the scale values of the parameter vary between −0.2 and 0.4. Here, DPH fluorescence anisotropy changes from 0.14 to 0.06 for POPC and SOPC vesicles in water (Figure 6 and Figure 7), thus indicating membranes in liquid-disordered phase in the temperature range studied from 20 to 60 °C. This representation allows for discerning the different trends in the thermotropic behavior of POPC and SOPC bilayers in glucose and sucrose solutions.

Upon increasing temperature, DPH fluorescence anisotropy is reduced both in POPC and SOPC LUVs, which corresponds to an increase in membrane fluidity. For the two PC studied, we observe different behavior of $r_{DPH}$ upon addition of glucose and sucrose in the aqueous phase. While in glucose solutions, no correlation between DPH fluorescence anisotropy of POPC vesicles and the monosaccharide concentration is found, the presence of sucrose in the aqueous surroundings leads to a decrease in DPH rotational diffusion. As far as DPH anisotropy is inversely proportional to membrane fluidity, the above results correspond to the formation of more ordered liquid hydrocarbon region of POPC bilayers in the presence of sucrose compared to the control sample. In sucrose solutions with increasing the temperature the value of DPH fluorescence anisotropy for both lipid compositions decreases differently in comparison to $r_{DPH}$ reduction of the control sample in water upon heating (Figure 6d and Figure 7d). At 20 °C DPH anisotropy in POPC membranes is lower in glucose-containing solution than in water, which corresponds to higher membrane fluidity. Inverse effect of sucrose on $r_{DPH}$ is observed at higher temperatures corresponding to decreased rotational diffusion of the fluorophore (Figure 6c,d). For SOPC, the inverse picture is observed with higher fluorescence anisotropy at 20 up to 55 °C. Upon further heating, we measure increased fluidity of SOPC bilayers both in glucose and sucrose solutions compared to the control sample (Figure 7c,d).

**Figure 8:**
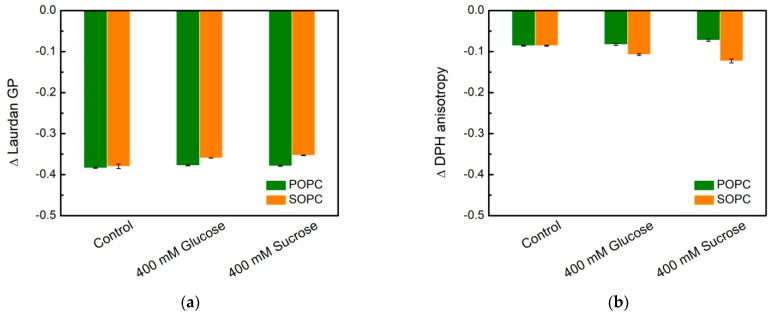
Lipid packing and membrane fluidity in single-component POPC and SOPC vesicles in water (controls) and in 400 mmol/L sugar solutions (glucose and sucrose): (**a**) ∆ Laurdan GP is defined as the difference between GP at 60 and 20 °C; (**b**) ∆ DPH anisotropy is defined as the difference between anisotropy at 60 and 20 °C. DPH anisotropy and Laurdan GP values represent the mean of three independent experiments. Error bars represent standard errors.

**Figure 9:**
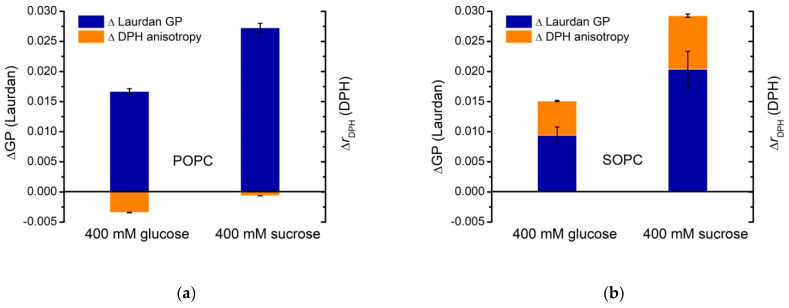
ΔGP and $\Delta r_{DPH}$ calculated as the difference between DPH anisotropy or Laurdan GP of (**a**) POPC and (**b**) SOPC vesicles in 400 mmol/L glucose or sucrose and in water (control) at 20 ℃. DPH anisotropy and Laurdan GP values represent the mean of three independent experiments. The error bars correspond to the standard deviations.

A noteworthy finding is the qualitatively different behavior of DPH anisotropy in POPC and SOPC bilayers as shown in Figure 8 and Figure 9. In Figure 8, fluorescence spectroscopy data for single-component POPC and SOPC vesicles in water and in 400 mmol/L sugar solutions (glucose and sucrose) are shown as the difference ∆ Laurdan GP between GP at 60 and 20 °C, and the difference ∆ DPH anisotropy between DPH fluorescence anisotropy at 60 and 20 °C, respectively. The change of lipid ordering and membrane fluidity of PC vesicles in water and sugar-containing solutions are displayed as the temperature increases. Sucrose solutions membranes (especially SOPC ones) are more thermostable compared to controls because of the smaller change between GP at 60 and 20 °C. Upon increasing the temperature, the effect of sucrose on the hydrophobic core fluidity is more pronounced for SOPC vesicles, while for POPC membranes, the fluidity alteration is weaker.

Figure 9 represents ΔGP and $\Delta r_{DPH}$, calculated as the difference between Laurdan generalized polarization and DPH anisotropy, respectively, measured for POPC vesicles with and without sugars in the bulk phase at 20℃. The same quantities are calculated also for SOPC LUVs (Figure 9b). The plot allows for discerning the different strength of the effect for the two types of PC studied in the presence of the mono- and disaccharide. The comparison between the reported values of $\Delta r_{DPH}$ and ΔGP suggests that the presence of glucose or sucrose in the aqueous surroundings affects the lipid packing in the bilayer more strongly at the glycerol level for both lipid compositions displayed in Figure 9. For SOPC bilayers in 400 mmol/L sucrose solutions, we obtain $\Delta GP/\Delta r_{DPH}\sim 2.3$ compared to ~1.7 at the same concentration of glucose. The effect is more pronounced for POPC samples yielding $\Delta GP/\Delta r_{DPH}\sim -43$ in sucrose-containing environment and ~−5 in glucose solutions, respectively. Here, the negative values reflect the slight reduction of DPH anisotropy in POPC membranes upon addition of 400 mmol/L glucose or sucrose in the aqueous surroundings (Figure 8 and Figure 9).

### 3.3. Dipole Potential in Lipid Bilayers and the Effect of Simple Carbohydrates

The fluorescence intensity ratio R_ex_ of di-8-ANEPPS dye (Equation (3)) in single-component lipid membranes composed of POPC and SOPC is measured in order to explore possible alterations in the primary hydration shell of the bilayer in the presence of sugars [51]. As discussed above, the measured value of R_ex_ is related to the electrical potential, which is generated transversally at the water-lipid interface and the hydrocarbon interior as a result of the contribution of polarized and polarizable chemical groups of lipid molecules and water surrounding the bilayer [36].

**Figure 10:**
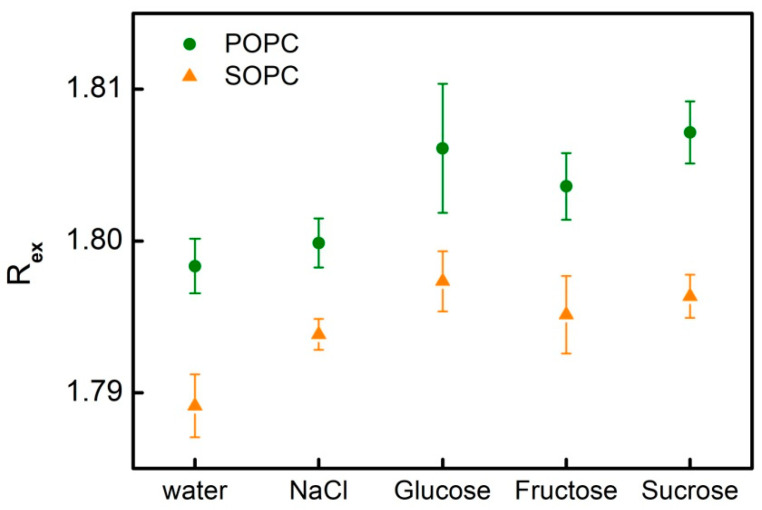
Di-8-ANEPPS fluorescence intensity ratio of POPC (circles) and SOPC (triangles) bilayers in 1 mmol/L NaCl sugar-free solutions and 200 mmol/L of glucose, fructose or sucrose; fluorescence excited at 420 and 520 nm and detected at 670 nm. Values are the mean of three independent experiments; the error bars correspond to the standard deviations.

For both types of phospholipids, we obtain an increase in di-8-ANEPPS fluorescence intensity ratio in 1 mmol/L NaCl and 200 mmol/L of the studied sugars compared to its value measured in water. The increase of R_ex_ stands for an increase of membrane dipole potential following from the interaction of solutes in the aqueous phase and the lipid bilayer. Slight alterations in membrane fluorescence intensity ratio are measured for POPC and SOPC bilayers upon the addition of 1 mmol/L NaCl and 200 mmol/L of glucose, fructose or sucrose (Table 3 and Figure 10).

The dipole potential measured here for POPC and SOPC membranes with and without sugars is consistent with the values obtained by other authors and methods in the range of 200–1000 mV for biomembranes [52,53]. The dipole potential of PC membranes is reported around 410 ± 150 mV in dependence of their degree of saturation, polar heads and the physicochemical properties of the buffer used. At low pH, as well as in the presence of molecules that increase the molecular order parameter and decrease the rotational order parameter, lipid membranes are susceptible to further increase of the dipole potential, similar to the effect of saturated PC compared to unsaturated PC.

## 4. Discussion

Both experimental methods applied in the present study for the measurement of the membrane electrical capacitance yield higher $C_{m}$ values at 200 mmol/L of sucrose in the aqueous surroundings compared to its value in the sugar-free control sample. The capacitance obtained from measurements on GUVs in sugar-free aqueous solutions $C_{m}=0.51\;\pm \;0.04\;\mu F/{\mathrm{cm}}^{2}$ is lower than values ~ 0.55÷1 $\mu F/{\mathrm{cm}}^{2}$ measured for planar bilayers of different lipid composition and charge [28,50,54,55,56,57] (data overview in Ref. [58]). In the present study, we confirm this trend by reporting higher specific electrical capacitance from experiments on Montal–Mueller BLMs compared to the values acquired from GUVs. Fluctuating vesicles are characterized by low-tension membranes [59,60], whereas for planar lipid bilayers the lateral tension is orders of magnitude higher [61,62]. Thus, a reduced membrane thickness of BLMs compared to GUVs is expected due to the tension difference for the two bilayer systems. Patch-clamp experiments on GUVs recently explored the bilayer thinning effect on membrane capacitance in 200 mmol/L sucrose and glucose solutions [58]. They have shown that membrane capacitance can vary with tension by up to 3%. The measurements reported in [58] have been performed in the relatively high-tension regime of mN/m. In the present study, we employed fluctuating free-standing vesicles with low membrane tensions, $10^{-6}÷10^{-4}$ mN/m as deduced from fluctuation spectroscopy [59,63,64,65]. Considering the vesicle electrodeformation involved in the capacitance measurements, here, membrane tensions are slightly elevated to $10^{-3}$ mN/m [39,66,67], which is orders of magnitude lower than the mN/m tensions applied in [58]. Despite the different tension range in [58], it can be concluded that membrane thinning associated to tension only partially explains the differences in the capacitance data acquired from GUVs and from planar lipid membranes. In our previous study [16] providing the first experimental evidence about the influence of sucrose on the electrical properties of lipid bilayers, we explored the possibility of changes in membrane dielectric constant as a factor affecting the capacitance. Here, we relate the reported increase of the electric capacitance of the bilayer and the corresponding increase in its dielectric permittivity [16] in the presence of ≥200 mmol/L sucrose to possible alterations in membrane structure and organization induced by membrane-sugar interactions [64].

The phosphatidylcholines POPC and SOPC studied here are synthetic mixed-acyl glycerophospholipids. The monounsaturated oleic acid residue (18:1) is positioned identically in both lipids (sn-2 position), while the saturated hydrocarbon chain at sn-1 position is different, in palmitic and stearic acid for POPC and SOPC, respectively. POPC bilayers undergo a gel-to-liquid crystalline phase transition around −2 °C [65]. The main phase transition of SOPC membranes occurs around 7 °C [66].

The fluorescence spectroscopy of Laurdan and DPH provide information about the lipid packing at different levels in the lipid bilayer, near the glycerol backbone and the hydrophobic core, respectively. Laurdan responds to the degree of hydration at the glycerol level, while DPH fluorescence anisotropy corresponds to the rotational diffusion of the probe in the hydrophobic region of the bilayer. Considering that the lipid molecules studied here are not characterized by truncated chains, we apply interchangeably the terms ordering and packing [67,68].

The amphiphilic fluorophore Laurdan comprises a naphthalene residue linked by an ester bond (hydrophilic) and a chain of lauric fatty acid (hydrophobic). As a result, it inserts the membrane parallel to lipid molecules in a way that its naphthalene moiety is located at the glycerol backbone of the lipid molecule and more precisely, at the level of *sn*-1 carbonyl [69]. Upon excitation by UV-light, the dipole moment of the fluorescent moiety increases leading to reorientation of the surrounding solvent dipoles. The results presented above (Figure 6 and Figure 7) suggest that at high temperatures, membrane becomes more loosely packed, which imparts higher mobility and increased dipolar relaxation at membrane interface. The reported results indicate that at higher temperatures the Laurdan GP values decrease for all measured LUV suspension compositions. Our findings support the expected thermotropic behavior of the lipid bilayer, whose packing order decreases upon increasing the temperature.

Reorientations of *sn*-1 and *sn*-2 chains lead to conformational and hydration changes at the glycerol level related also to reorientation of the lipid headgroups. Laurdan position distribution is characterized by a certain width as well as its possible relocation upon excitation. Thereby, the fluorescence wavelength is related to the fluorophore location depth in membrane. Shorter wavelengths are emitted by Laurdan molecules positioned deeper within the lipid bilayer, while red-shifted emission (longer wavelengths) occur from fluorophores located closer to membrane-water interface. Hence, Laurdan experiments are able to distinguish POPC from SOPC lipid ordering. Our results support the hypothesis about sucrose ordering effect in membrane in the proximity of the glycerol backbone (Figure 5; blue-shifted Laurdan emission signal).

The depolarization of fluorescence has been recognized as a reliable parameter for characterization of dynamic features and thermotropic behavior of the hydrophobic regions of lipid membranes and lipoproteins [31]. Measurements of DPH fluorescence anisotropy allow evaluating the hindrance of the fluorophore mobility in the hydrophobic core of the bilayer related to alterations in packing of the aliphatic chains. We obtain that the increase of the sucrose content in the aqueous surrounding leads to hindering the DPH rotational diffusion as a result from to the formation of more ordered liquid phase.

The relative changes, $\Delta r_{DPH}$ and ΔGP, state that the reduction in rotational diffusion and degree of hydration for the corresponding molecular probe is larger in the presence of the disaccharide. This reduction is more considerable for Laurdan (Figure 9). Therefore, the changes in lipid ordering induced by the presence of sucrose are predominantly at the glycerol level rather than in the hydrophobic core.

In order to further elucidate the effect of sucrose binding [64,70] on the electric properties of lipid bilayers, we study the bilayer dipole potential, which occurs due to the hydrated polar headgroups, the glycerol-ester region of the lipids and the functional group dipoles of the terminal methyl groups of hydrocarbon chains. This membrane parameter is still limitedly understood but undoubtedly recognized as an important regulator of membrane structure and function [71]. Numerous examples can be given in this respect such as the modulation of the hydration force interplaying in membrane–membrane and membrane-ligand interactions or the lipid-mediated cellular signaling in cells. The dipole potential arises in a medium over which the dielectric constant is changing in a large interval—from 2 to 80 [71]. Even if the microscopic nature of the interactions leading to the creation of the dipole potential remains not completely described, some elaborated theoretical models of its origin account for the important role of interfacial water molecules.

The slight increase in the dipole potential reported here for POPC and SOPC bilayers upon the addition of sodium chloride and small carbohydrates is in agreement with the trend measured for monolayers of dimyristoylphosphatidylcholine on the air/water interface at 20 °C, which is below the main transition temperature of the lipid [51]. Four sucrose molecules have been reported to displace three water molecules per lipid, thus producing only a weak effect on the dipole potential or the carbonyl groups in monolayers [51]. In contrast to trehalose, sucrose has not been found to interact directly with the phospholipid groups, thus implying that this disaccharide is not expected to replace water molecules in the tightly bound hydration sphere. Upon increasing the sugar concentration, the water activity in the bulk solution decreases [72]. Furthermore, the refractive index changes observed in vesicles under osmotic stress inferred alterations in the extent of hydration and/or lipid packing of phospholipid molecules [73]. Hence, sucrose would exhibit a colligative effect on fully hydrated bilayers by osmotically extruding water from the bilayer.

Our findings suggest possible alterations of the bilayer relative permittivity in sucrose-containing aqueous surroundings. They are coherent with our previous results for sucrose solutions with high ionic strength [16]. From the capacitance data and the reported membrane thickness [64], the values of the relative dielectric permittivity have been evaluated to vary from ~2.3 (sugar-free, 10 mmol/L NaCl) to ~3.5 (for sucrose concentrations above ~200 mmol/L and 10 mmol/L NaCl). Following the approach here, we estimate a narrower range of $\varepsilon_{rm}$ changes upon increasing sucrose content in the bulk phase, characterized by an order of magnitude of lower ionic strength. POPC bilayer relative permittivity varies from $\varepsilon_{rm}\sim$2.4 in 1 mM NaCl up to $\varepsilon_{rm}\sim$2.9 in the presence of sucrose with concentrations >200 mmol/L in the electrolyte solution.

The above evaluations for the relative dielectric permittivity are performed considering the integral thickness of the bilayer, including its hydrophobic part and headgroup regions. In order to appropriately account for the contribution of the hydrocarbon core and the headgroup regions, the bilayer has to be described by an equivalent circuit of capacitors in series replacing $C_{m}$ in Equation (5). Attempts to analyze the obtained alteration of $\varepsilon_{m}$ are based on the data available in the literature for the thickness of the headgroup region of PC ~ 9 Å [74] and the thickness of dioleoylphosphatidylcholine (DOPC) bilayers in sugar-containing aqueous solutions concentrations [64]. This approximation is realistic as the head groups of DOPC and POPC are identical and both lipids are characterized by similar hydrophobic lengths. The hydrophobic thickness of DOPC and POPC is reported to be between 27.1 and 27.2 Å [75,76] for the former, and 27.1 Å [77] for the latter. Estimations for the relative dielectric permittivity of the dipolar region of phosphatidylcholine lipid bilayers have given a wide range of values depending on the theoretical model applied [78]. The consideration of the rotating polar headgroups as an ensemble of interacting dipoles embedded in a nonhomogeneous dielectric with explicitly considering the interactions between the nearest neighborhood has derived ~30 for the headgroup dielectric constant [78], thus providing for the capacitance of the headgroup region a value of the order of 15 µF/cm^2^. As shown by Velikonja et al. [44] at high monovalent salt concentrations the relative permittivity in the dipolar headgroup region is decreased due to a saturation effect in orientational ordering of water dipoles. Considering that our study is performed at nearly 500 times lower salt concentrations we hypothesize that the changes in the dielectric properties are much more pronounced in the hydrophobic core than in the dipolar head region. In this case, one should keep in mind that further theoretical investigations could help quantifying the sucrose effect on the dielectric permittivity of the headgroup region. Here, we suppose that the obtained alteration of $\varepsilon_{m}$ is a result mainly of modulation in the hydrocarbon core of the bilayer. Hence, we deduce that in the presence of high sucrose concentrations (≥200 mmol/L) the relative dielectric permittivity of hydrocarbon chains is increased by ~14% and varies between ~1.4 and 1.6 in the studied sucrose concentration range. Considering the narrower range of $\varepsilon_{rm}$ changes upon increasing sucrose content in the bulk phase at an order of magnitude lower ionic strength than in [16] we formulate a hypothesis for a cooperative effect of sodium chloride and sucrose on membrane properties, including the bilayer specific capacitance and relative permittivity. It is supported by previous results indicating that the presence of sodium chloride modifies the effect of sucrose on the bending rigidity of lipid bilayers. We found that membrane bending elasticity measured in sucrose solutions containing 10 mM NaCl was independent of the disaccharide concentration in the aqueous surroundings [79]. This result has to be considered in the light of the ion-induced modification of sucrose-phosphatidylcholine hydrogen bond network, reported in the literature [80,81]. The capability of disaccharides to replace water molecules [51] and to create a water-like hydrogen bond network in the lipid surroundings contributes to retaining of the molecular properties of lipids [6]. Molecular dynamics simulation results have provided evidence that the hydrogen bond network of phosphatidylcholines and sucrose is partially disrupted in the presence of sodium and chloride ions [81].

## 5. Conclusions

The active research in the field of membrane biophysics deepens the understanding of the structural and functional membrane features in regard to vital processes in cells. In the present study a step forward is performed toward revealing the impact of small carbohydrates on the bilayer electrical properties, structure and organization. Modulation of membrane dielectric properties (capacitance and relative permittivity) is reported at moderate concentrations (above 200 mmol/L) of sucrose in the bulk phase. The above parameters are insensitive to the presence of fructose or glucose in the aqueous solution. A cooperative effect of sodium chloride and sucrose on membrane properties is suggested. As obtained by FFT EIS, all three of the small carbohydrate molecules studied here contribute to increased bilayer electric resistance. A qualitatively different behavior of the hydrocarbon fluidity in the two types of phosphatidylcholine bilayers, POPC and SOPC, upon the addition of sucrose in the aqueous surroundings is found. The hypothesis about sucrose ordering effect in membrane in the proximity of the glycerol backbone is supported here. The reduction in rotational diffusion and degree of hydration for the corresponding fluorophore is larger in the presence of the disaccharide studied. Sucrose is found to induce larger changes in lipid ordering at the glycerol level rather than in the hydrophobic core. A slight increase in the dipole potential is reported for POPC and SOPC bilayers upon the addition of sodium chloride, glucose, fructose and sucrose. The presented results are expected to be useful for the appropriate choice of carbohydrates, when the efficiency of the application targets the preservation of membrane electric properties. Gaining knowledge of the modulation of membrane molecular organization and its electric characteristics (capacitance, dielectric permittivity and resistance) by small carbohydrates would foster the elaboration of sugar-based lipid biomimetic systems for electric field-assisted applications in food industry, biotechnology and medicine.

## Figures and Tables

**Table 1:** Specific capacitance of POPC membranes in aqueous solutions containing different concentrations up to 300 mmol/L glucose, fructose or sucrose; data obtained from electrodeformation of GUVs; GF, goodness of fit. The errors in ${\bar{C}}_{m}$ and $C_{m}$ are standard deviations.

Sugar, mmol/L	λ*_in_*, µS/cm	Λ	${\bar{C}}_{m},\;µF/{\mathrm{cm}}^{2}$ (Number of Vesicles)	$C_{m},\;µF/{\mathrm{cm}}^{2}$	GF
Control					
0	258	0.87	0.44 ± 0.03 (7)	0.51 ± 0.04	0.75
Sucrose					
50	276	0.95	0.45 ± 0.02 (9)	0.53 ± 0.03	0.73
100	238	0.93	0.49 ± 0.04 (11)	0.59 ± 0.05	0.44
200	363	0.95	0.53 ± 0.03 (10)	0.65 ± 0.04	0.82
300	347	0.94	0.54 ± 0.03 (8)	0.66 ± 0.04	0.84
Glucose					
50	318	0.94	0.45 ± 0.04 (8)	0.53 ± 0.05	0.93
100	257	0.88	0.45 ± 0.05 (6)	0.53 ± 0.06	0.66
200	192	0.88	0.45 ± 0.05 (6)	0.53 ± 0.07	0.31
300	223	0.90	0.44 ± 0.02 (8)	0.52 ± 0.02	0.96
Fructose					
50	145	0.88	0.47 ± 0.04 (15)	0.56 ± 0.04	0.53
100	312	0.95	0.43 ± 0.02 (7)	0.50 ± 0.02	0.48
200	148	0.87	0.42 ± 0.02 (10)	0.49 ± 0.02	0.80
300	145	0.87	0.45 ± 0.04 (10)	0.55 ± 0.04	0.64

**Table 2:** Specific capacitance and resistance of POPC membranes in aqueous solutions containing 1 mmol/L NaCl and 200 mmol/L glucose, fructose or sucrose; data obtained from FFT-EIS of BLMs by equivalent model circuit fits; control samples contain only 1 mmol/L NaCl.

Sugar	$R_{m},\;10^{6}\;\Omega \;{\mathrm{cm}}^{2}$	$C_{m},\;µF/{\mathrm{cm}}^{2}$	Number of Samples	GF
Control	1.42 ± 0.05	0.79 ± 0.06	7	0.51
Glucose	1.62 ± 0.08	0.85 ± 0.07	4	0.16
Fructose	1.57 ± 0.06	0.87 ± 0.05	7	0.71
Sucrose	1.56 ± 0.02	1.10 ± 0.10	6	0.43

**Table 3:** Di-8-ANEPPS fluorescence intensity ratio and dipole potential of POPC bilayers in aqueous solutions containing 1 mmol/L NaCl and 200 mmol/L glucose, fructose or sucrose; control samples are measured in bidistilled water and in 1 mmol/L NaCl; fluorescence excited at 420 and 520 nm and detected at 670 nm.

Sample	POPC		SOPC	
	R_ex_	$\Psi_{d}\;(\mathrm{mV})$	R_ex_	$\Psi_{d}\;(\mathrm{mV})$
H_2_O, bidistilled	1.798 ± 0.002	488	1.789 ± 0.002	486
1 mM NaCl	1.800 ± 0.002	488	1.794 ± 0.001	487
200 mM Glucose, 1 mM NaCl	1.806 ± 0.004	490	1.797 ± 0.002	488
200 mM Fructose, 1 mM NaCl	1.804 ± 0.002	489	1.795 ± 0.003	487
200 mM Sucrose, 1 mM NaCl	1.807 ± 0.002	490	1.796 ± 0.001	487

## Data Availability

Data is contained within the article.

## Abbreviations

BLM	bilayer lipid membrane
Di-8-ANEPPS	4-(2-[6-(Dioctylamino)-2-naphthalenyl]ethenyl)-1-(3-sulfopropyl) pyridinium inner salt
DPH	1,6-diphenyl-1,3,5-hexatriene
FFT-EIS	fast Fourier transform electrochemical impedance spectroscopy
GP	generalized polarization
GUV	giant unilamellar vesicle
ITO	indium tin oxide
Laurdan	6-dodecanoyl-N, N-dimethyl-2-naphthylamine
LUV	large unilamellar vesicle
PC	phosphatidylcholine
PDMS	polydimethylsiloxane
POPC	1-palmitoyl-2-oleoyl-*sn*-glycero-3-phosphocholine
SOPC	1-stearoyl-2-oleoyl-*sn*-glycero-3-phosphocholine
